# *N*′-[(*E*)-4-Chloro­benzyl­idene]pyridine-4-carbohydrazide monohydrate

**DOI:** 10.1107/S1600536812029121

**Published:** 2012-06-30

**Authors:** Hoong-Kun Fun, Wan-Sin Loh, Divya N. Shetty, B. Narayana, B. K. Sarojini

**Affiliations:** a X-ray Crystallography Unit, School of Physics, Universiti Sains Malaysia, 11800 USM, Penang, Malaysia; b Department of Studies in Chemistry, Mangalore University, Mangalagangotri 574 199, India; c Department of Chemistry, P.A. College of Engineering, Nadupadavu, Mangalore 574 153, India

## Abstract

The asymmetric unit of the title compound, C_13_H_10_ClN_3_O·H_2_O, consists of two crystallographically independent Schiff base mol­ecules which exist in an *E* conformation with respect to the C=N double bond, and two independent water mol­ecules. In the crystal, the Schiff base and water mol­ecules are linked into a three-dimensional network *via* N—H⋯O, O—H⋯N, O—H⋯O and C—H⋯O hydrogen bonds. The crystal studied was a pseudo-merohedral twin with twin law (101 0-10 00-1) and a component ratio of 0.792 (2):0.208 (2).

## Related literature

For background to terphenyls, see: Naveenkumar *et al.* (2010 ▶); Chen (2006 ▶). For related structures, see: Fun, Quah, Shetty *et al.* (2012 ▶); Fun, Quah, Shyma *et al.* (2012 ▶). For the stability of the temperature controller used in the data collection, see: Cosier & Glazer (1986 ▶).
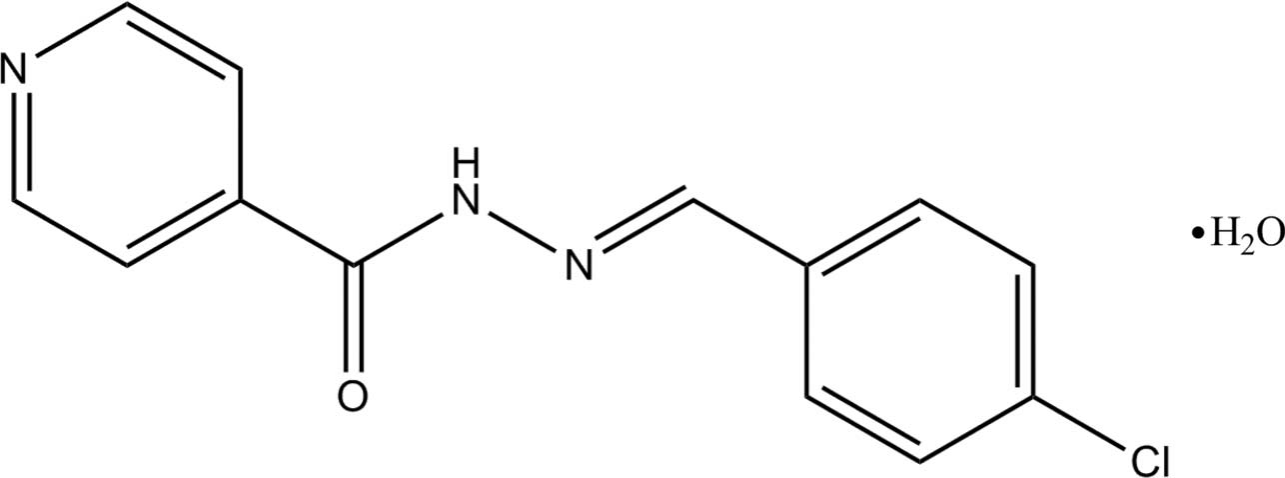


## Experimental

### 

#### Crystal data

C_13_H_10_ClN_3_O·H_2_O
*M* *_r_* = 277.71
Monoclinic, 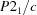

*a* = 14.1645 (7) Å
*b* = 14.6276 (7) Å
*c* = 14.0817 (7) Å
β = 119.220 (2)°
*V* = 2546.4 (2) Å^3^
*Z* = 8
Mo *K*α radiation
μ = 0.30 mm^−1^
*T* = 100 K
0.47 × 0.26 × 0.24 mm

#### Data collection

Bruker SMART APEXII CCD area-detector diffractometer
Absorption correction: multi-scan (*SADABS*; Bruker, 2009 ▶) *T*_min_ = 0.871, *T*_max_ = 0.931
20683 measured reflections
4458 independent reflections
3995 reflections with *I* > 2σ(*I*)
*R*_int_ = 0.043

#### Refinement

*R*[*F* ^2^ > 2σ(*F* ^2^)] = 0.071
*wR*(*F* ^2^) = 0.205
*S* = 1.06
4458 reflections
360 parameters
H atoms treated by a mixture of independent and constrained refinement
Δρ_max_ = 0.80 e Å^−3^
Δρ_min_ = −0.55 e Å^−3^

### 

Data collection: *APEX2* (Bruker, 2009 ▶); cell refinement: *SAINT* (Bruker, 2009 ▶); data reduction: *SAINT*; program(s) used to solve structure: *SHELXTL* (Sheldrick, 2008 ▶); program(s) used to refine structure: *SHELXTL*; molecular graphics: *SHELXTL*; software used to prepare material for publication: *SHELXTL* and *PLATON* (Spek, 2009 ▶).

## Supplementary Material

Crystal structure: contains datablock(s) global, I. DOI: 10.1107/S1600536812029121/is5159sup1.cif

Structure factors: contains datablock(s) I. DOI: 10.1107/S1600536812029121/is5159Isup2.hkl

Supplementary material file. DOI: 10.1107/S1600536812029121/is5159Isup3.cml

Additional supplementary materials:  crystallographic information; 3D view; checkCIF report

## Figures and Tables

**Table 1:** Hydrogen-bond geometry (Å, °)

*D*—H⋯*A*	*D*—H	H⋯*A*	*D*⋯*A*	*D*—H⋯*A*
N2*A*—H1*NA*⋯O1*WA*	1.00	1.88	2.838 (7)	160
N2*B*—H1*NB*⋯O1*WB* ^i^	0.89	1.95	2.810 (7)	161
O1*WA*—H1*WA*⋯N1*A* ^ii^	0.88 (9)	2.14 (8)	2.896 (7)	144 (6)
O1*WA*—H2*WA*⋯O1*B* ^iii^	0.86 (10)	2.05 (9)	2.817 (6)	149 (9)
O1*WA*—H2*WA*⋯N3*B* ^iii^	0.86 (10)	2.59 (10)	3.306 (6)	142 (8)
O1*WB*—H2*WB*⋯O1*A* ^iv^	0.73 (9)	2.19 (8)	2.843 (6)	150 (8)
O1*WB*—H1*WB*⋯N1*B* ^iv^	0.81 (9)	2.00 (9)	2.798 (6)	166 (11)
C1*A*—H1*AA*⋯O1*WA*	0.95	2.49	3.321 (6)	146
C1*A*—H1*AA*⋯O1*B* ^iii^	0.95	2.54	3.277 (7)	135
C7*A*—H7*AA*⋯O1*WA*	0.95	2.46	3.247 (7)	141
C1*B*—H1*BA*⋯O1*WB* ^i^	0.95	2.43	3.201 (7)	138
C1*B*—H1*BA*⋯O1*A* ^v^	0.95	2.52	3.230 (8)	131

Symmetry codes: (i) 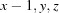; (ii) 
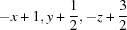
; (iii) 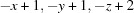; (iv) 
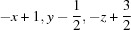
; (v) 
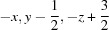
.

**Fig. 1.**
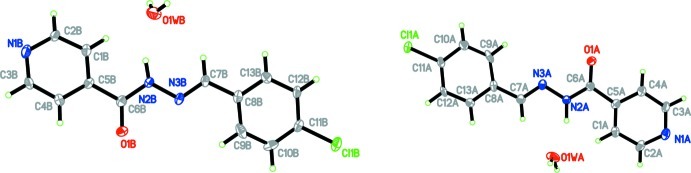
The molecular structure of the title compound, showing 50% probability displacement ellipsoids.

**Fig. 2.**
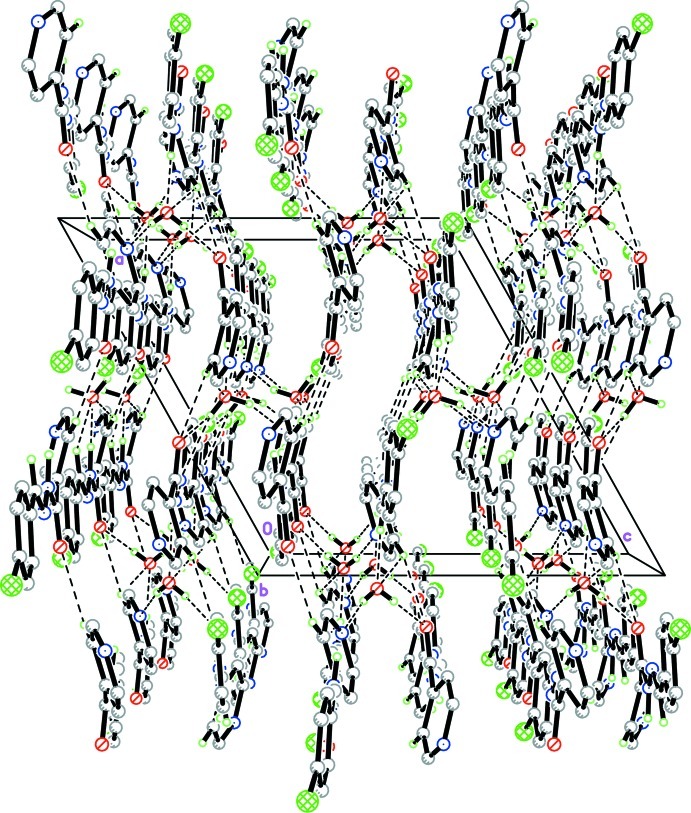
The crystal packing of the title compound, viewed along the b axis. H atoms not involved in the intermolecular interactions (dashed lines) have been omitted for clarity.

## supplementary crystallographic information

### Comment

The pharmaceutical importance of isoniazid and its various derivatives are well documented and also the crystal structures of its Schiff base derivatives have been reported (Naveenkumar *et al.*, 2010; Chen, 2006). Hence, we report herein the synthesis and crystal structure of title compound. The Schiff base, *N*'-[(*E*)-(4-chlorophenyl)methylidene] pyridine-4-carbohydrazide, is synthesized by condensation of isoniazid with 4-chlorobenzaldehyde in absolute alcohol in presence of hydrochloric acid. The Schiff base crystallized out as a hydrate to form the title compound.

The asymmetric unit of the Schiff base compound, (Fig. 1), consists of two crystallographically independent *N*'-[(*E*)-(4-chlorophenyl)methylidene]pyridine-4-carbohydrazide molecules and two water molecules. The Schiff base molecules exist in an *E* configuration with respect to the C7A═N3A and C7B═N3B double bonds. The pyridine rings (C1A/C2A/N1A/C3A/C4A/C5A & C1B/C2B/N1B/C3B/C4B/C5B) are approximately planar with maximum deviations of 0.016 (6) Å at atom C4A and 0.012 (5) Å at atom C6B. Bond lengths and angles are within the normal ranges and are comparable with the related structures (Fun, Quah, Shetty *et al.*, 2012; Fun, Quah, Shyma *et al.*, 2012).

In the crystal packing (Fig. 2), the molecules are linked into a three-dimensional network *via* intermolecular N2A—H1NA···O1WA, N2B—H1NB···O1WB, O1WA—H1WA···N1A, O1WA—H2WA···O1B, O1WA—H2WA···N3B, O1WB—H2WB···O1A, C1A—H1AA···O1WA, C1A—H1AA···O1B, C7A—H7AA···O1WA, C1B—H1BA···O1WB, O1WB—H1WB···N1B and C1B—H1BA···O1A hydrogen bonds (Table 1).

### Experimental

A mixture of isoniazid (1.4 g, 0.01 mol) and 4-chlorobenzaldehyde (1.4 g, 0.01 mol) in 15 ml of absolute alcohol containing 2 drops of hydrochloric acid was refluxed for about 3 h. Upon cooling, the solid was separated and was filtered and recrystallized from DMF. The Schiff base compound was crystallized out as a hydrate by slow evaporation in DMF. *M.P.*: 489 K.

### Refinement

The N- and O-bound H atoms were located from a difference Fourier map. The O-bound H atoms were refined freely, whereas the N-bound H atoms were refined with a riding model with *U*_iso_(H) = 1.5 *U*_eq_(N) [O—H = 0.73 (7) to 0.89 (8) Å; N—H = 0.89 and 1.00 Å]. The remaining H atoms were positioned geometrically and were refined with a riding model with *U*_iso_(H) = 1.2 *U*_eq_(C). The crystal studied was a twin with twin law, 101 010 001 and BASF = 0.208 (2).

### Crystal data

**Table:**

C_13_H_10_ClN_3_O·H_2_O	*F*(000) = 1152
*M**_r_* = 277.71	*D*_x_ = 1.449 Mg m^−^^3^
Monoclinic, *P*2_1_/*c*	Mo *K*α radiation, λ = 0.71073 Å
Hall symbol: -P 2ybc	Cell parameters from 8764 reflections
*a* = 14.1645 (7) Å	θ = 2.8–29.9°
*b* = 14.6276 (7) Å	µ = 0.30 mm^−^^1^
*c* = 14.0817 (7) Å	*T* = 100 K
β = 119.220 (2)°	Block, yellow
*V* = 2546.4 (2) Å^3^	0.47 × 0.26 × 0.24 mm
*Z* = 8	

### Data collection

**Table:**

Bruker SMART APEXII CCD area-detector diffractometer	4458 independent reflections
Radiation source: fine-focus sealed tube	3995 reflections with *I* > 2σ(*I*)
Graphite monochromator	*R*_int_ = 0.043
φ and ω scans	θ_max_ = 25.0°, θ_min_ = 1.4°
Absorption correction: multi-scan (*SADABS*; Bruker, 2009)	*h* = −16→16
*T*_min_ = 0.871, *T*_max_ = 0.931	*k* = −17→13
20683 measured reflections	*l* = −16→16

### Refinement

**Table:**

Refinement on *F*^2^	Primary atom site location: structure-invariant direct methods
Least-squares matrix: full	Secondary atom site location: difference Fourier map
*R*[*F*^2^ > 2σ(*F*^2^)] = 0.071	Hydrogen site location: inferred from neighbouring sites
*wR*(*F*^2^) = 0.205	H atoms treated by a mixture of independent and constrained refinement
*S* = 1.06	*w* = 1/[σ^2^(*F*_o_^2^) + (0.123*P*)^2^ + 5.736*P*] where *P* = (*F*_o_^2^ + 2*F*_c_^2^)/3
4458 reflections	(Δ/σ)_max_ = 0.001
360 parameters	Δρ_max_ = 0.80 e Å^−^^3^
0 restraints	Δρ_min_ = −0.55 e Å^−^^3^

### Special details

**Table:**

Experimental. The crystal was placed in the cold stream of an Oxford Cryosystems Cobra open-flow nitrogen cryostat (Cosier & Glazer, 1986) operating at 100.0 (1) K.
Geometry. All e.s.d.'s (except the e.s.d. in the dihedral angle between two l.s. planes) are estimated using the full covariance matrix. The cell e.s.d.'s are taken into account individually in the estimation of e.s.d.'s in distances, angles and torsion angles; correlations between e.s.d.'s in cell parameters are only used when they are defined by crystal symmetry. An approximate (isotropic) treatment of cell e.s.d.'s is used for estimating e.s.d.'s involving l.s. planes.
Refinement. Refinement of *F*^2^ against ALL reflections. The weighted *R*-factor *wR* and goodness of fit *S* are based on *F*^2^, conventional *R*-factors *R* are based on *F*, with *F* set to zero for negative *F*^2^. The threshold expression of *F*^2^ > σ(*F*^2^) is used only for calculating *R*-factors(gt) *etc*. and is not relevant to the choice of reflections for refinement. *R*-factors based on *F*^2^ are statistically about twice as large as those based on *F*, and *R*- factors based on ALL data will be even larger.

### Fractional atomic coordinates and isotropic or equivalent isotropic displacement parameters (Å^2^)

**Table:**

	*x*	*y*	*z*	*U*_iso_*/*U*_eq_	
Cl1A	0.07219 (10)	1.37835 (8)	0.59204 (11)	0.0295 (3)	
O1A	0.1241 (3)	0.7654 (2)	0.6526 (3)	0.0223 (7)	
N1A	0.4094 (3)	0.5253 (3)	0.7583 (3)	0.0237 (9)	
H1NA	0.3567	0.8519	0.7510	0.035*	
N2A	0.2758 (3)	0.8520 (3)	0.7089 (3)	0.0191 (8)	
H1NB	0.1442	0.1632	0.8694	0.029*	
N3A	0.2166 (3)	0.9323 (3)	0.6802 (3)	0.0206 (8)	
C1A	0.4010 (4)	0.6845 (3)	0.7977 (4)	0.0222 (10)	
H1AA	0.4371	0.7375	0.8380	0.027*	
C2A	0.4562 (4)	0.6018 (3)	0.8149 (4)	0.0247 (11)	
H2AA	0.5303	0.5994	0.8690	0.030*	
C3A	0.3042 (4)	0.5298 (3)	0.6855 (4)	0.0233 (10)	
H3AA	0.2700	0.4761	0.6454	0.028*	
C4A	0.2420 (4)	0.6080 (3)	0.6647 (4)	0.0228 (10)	
H4AA	0.1669	0.6073	0.6138	0.027*	
C5A	0.2924 (4)	0.6874 (3)	0.7204 (4)	0.0188 (10)	
C6A	0.2226 (4)	0.7718 (3)	0.6916 (4)	0.0179 (10)	
C7A	0.2727 (4)	1.0055 (3)	0.6964 (4)	0.0213 (10)	
H7AA	0.3488	1.0011	0.7256	0.026*	
C8A	0.2212 (4)	1.0954 (3)	0.6706 (4)	0.0208 (10)	
C9A	0.1071 (4)	1.1091 (3)	0.6206 (4)	0.0225 (11)	
H9AA	0.0609	1.0574	0.6028	0.027*	
C10A	0.0632 (4)	1.1930 (3)	0.5980 (4)	0.0230 (10)	
H10A	−0.0130	1.2005	0.5646	0.028*	
C11A	0.1303 (4)	1.2690 (3)	0.6241 (4)	0.0192 (10)	
C12A	0.2426 (4)	1.2607 (3)	0.6729 (4)	0.0223 (10)	
H12A	0.2875	1.3132	0.6904	0.027*	
C13A	0.2864 (4)	1.1740 (3)	0.6951 (4)	0.0220 (10)	
H13A	0.3627	1.1671	0.7278	0.026*	
O1WA	0.4993 (3)	0.8952 (3)	0.8295 (4)	0.0354 (9)	
Cl1B	0.42027 (11)	−0.36679 (8)	1.05982 (11)	0.0290 (3)	
O1B	0.3756 (3)	0.2301 (2)	1.0041 (3)	0.0270 (8)	
N1B	0.1149 (3)	0.4890 (3)	0.8468 (3)	0.0241 (9)	
N2B	0.2152 (3)	0.1541 (3)	0.9120 (3)	0.0188 (8)	
N3B	0.2689 (3)	0.0713 (3)	0.9449 (3)	0.0225 (9)	
C1B	0.1096 (4)	0.3298 (3)	0.8844 (4)	0.0210 (10)	
H1BA	0.0696	0.2791	0.8881	0.025*	
C2B	0.0618 (4)	0.4152 (3)	0.8538 (4)	0.0211 (10)	
H2BA	−0.0114	0.4220	0.8371	0.025*	
C3B	0.2179 (4)	0.4770 (3)	0.8718 (4)	0.0231 (10)	
H3BA	0.2562	0.5286	0.8673	0.028*	
C4B	0.2721 (4)	0.3956 (3)	0.9034 (4)	0.0225 (10)	
H4BA	0.3457	0.3915	0.9205	0.027*	
C5B	0.2176 (4)	0.3195 (3)	0.9099 (4)	0.0192 (10)	
C6B	0.2767 (4)	0.2306 (3)	0.9467 (4)	0.0179 (10)	
C7B	0.2105 (4)	−0.0009 (3)	0.9066 (4)	0.0214 (10)	
H7BA	0.1351	0.0042	0.8576	0.026*	
C8B	0.2620 (4)	−0.0915 (3)	0.9397 (4)	0.0213 (10)	
C9B	0.3751 (4)	−0.1005 (4)	1.0003 (4)	0.0273 (11)	
H9BA	0.4189	−0.0473	1.0168	0.033*	
C10B	0.4230 (4)	−0.1829 (4)	1.0357 (4)	0.0258 (11)	
H10B	0.4995	−0.1876	1.0767	0.031*	
C11B	0.3585 (4)	−0.2605 (3)	1.0112 (4)	0.0202 (10)	
C12B	0.2471 (4)	−0.2557 (3)	0.9494 (4)	0.0202 (10)	
H12B	0.2042	−0.3095	0.9315	0.024*	
C13B	0.1991 (4)	−0.1706 (3)	0.9140 (4)	0.0219 (10)	
H13B	0.1226	−0.1661	0.8719	0.026*	
O1WB	0.9892 (3)	0.1399 (3)	0.7853 (4)	0.0367 (10)	
H1WA	0.520 (6)	0.914 (5)	0.783 (6)	0.05 (2)*	
H2WA	0.551 (7)	0.876 (6)	0.890 (8)	0.08 (3)*	
H1WB	0.960 (7)	0.101 (6)	0.739 (7)	0.06 (3)*	
H2WB	0.955 (6)	0.155 (5)	0.808 (6)	0.04 (2)*	

### Atomic displacement parameters (Å^2^)

**Table:**

	*U*^11^	*U*^22^	*U*^33^	*U*^12^	*U*^13^	*U*^23^
Cl1A	0.0284 (7)	0.0147 (6)	0.0418 (8)	0.0036 (5)	0.0143 (6)	−0.0003 (5)
O1A	0.0173 (16)	0.0182 (18)	0.0292 (18)	0.0014 (13)	0.0097 (14)	0.0030 (14)
N1A	0.031 (2)	0.017 (2)	0.029 (2)	0.0044 (17)	0.0186 (19)	0.0044 (17)
N2A	0.0169 (19)	0.0136 (19)	0.025 (2)	0.0003 (16)	0.0090 (17)	0.0039 (16)
N3A	0.023 (2)	0.014 (2)	0.024 (2)	0.0019 (16)	0.0108 (17)	0.0050 (16)
C1A	0.028 (3)	0.015 (2)	0.023 (2)	0.000 (2)	0.013 (2)	0.0009 (19)
C2A	0.029 (3)	0.020 (2)	0.024 (2)	0.008 (2)	0.012 (2)	0.004 (2)
C3A	0.028 (3)	0.015 (2)	0.032 (3)	0.003 (2)	0.018 (2)	0.000 (2)
C4A	0.024 (3)	0.018 (2)	0.026 (3)	0.003 (2)	0.011 (2)	0.002 (2)
C5A	0.021 (2)	0.017 (2)	0.023 (2)	0.0025 (19)	0.0151 (19)	0.0068 (19)
C6A	0.020 (2)	0.016 (2)	0.019 (2)	0.0012 (18)	0.0097 (19)	0.0030 (18)
C7A	0.020 (2)	0.017 (2)	0.024 (2)	0.0002 (19)	0.009 (2)	0.004 (2)
C8A	0.031 (3)	0.012 (2)	0.021 (2)	−0.002 (2)	0.014 (2)	−0.0017 (19)
C9A	0.036 (3)	0.017 (2)	0.016 (2)	−0.014 (2)	0.013 (2)	−0.0015 (18)
C10A	0.023 (2)	0.023 (3)	0.024 (2)	−0.003 (2)	0.012 (2)	−0.004 (2)
C11A	0.024 (2)	0.014 (2)	0.021 (2)	−0.0035 (18)	0.013 (2)	−0.0058 (18)
C12A	0.025 (2)	0.011 (2)	0.030 (3)	−0.0074 (19)	0.013 (2)	−0.0045 (19)
C13A	0.017 (2)	0.018 (2)	0.027 (2)	−0.0012 (19)	0.007 (2)	0.000 (2)
O1WA	0.0234 (19)	0.034 (2)	0.038 (2)	−0.0067 (17)	0.0060 (18)	0.0152 (18)
Cl1B	0.0340 (7)	0.0176 (6)	0.0350 (7)	0.0075 (5)	0.0164 (6)	0.0055 (5)
O1B	0.0162 (17)	0.024 (2)	0.0332 (19)	0.0029 (14)	0.0061 (15)	0.0080 (15)
N1B	0.029 (2)	0.015 (2)	0.026 (2)	0.0047 (17)	0.0112 (18)	0.0035 (16)
N2B	0.0162 (19)	0.0140 (19)	0.022 (2)	0.0025 (16)	0.0062 (16)	0.0010 (16)
N3B	0.023 (2)	0.013 (2)	0.031 (2)	0.0042 (17)	0.0123 (18)	−0.0022 (17)
C1B	0.025 (2)	0.016 (2)	0.022 (2)	0.0002 (19)	0.011 (2)	0.0015 (19)
C2B	0.021 (2)	0.015 (2)	0.024 (2)	0.0032 (19)	0.009 (2)	0.0004 (19)
C3B	0.025 (3)	0.015 (2)	0.028 (3)	−0.004 (2)	0.011 (2)	0.002 (2)
C4B	0.023 (2)	0.017 (2)	0.027 (2)	−0.002 (2)	0.012 (2)	0.000 (2)
C5B	0.019 (2)	0.018 (2)	0.019 (2)	0.0040 (19)	0.0082 (19)	0.0029 (19)
C6B	0.022 (2)	0.019 (3)	0.015 (2)	0.0032 (19)	0.0102 (19)	0.0037 (18)
C7B	0.023 (2)	0.013 (2)	0.027 (2)	0.0012 (19)	0.011 (2)	−0.0044 (19)
C8B	0.028 (3)	0.015 (2)	0.023 (2)	0.002 (2)	0.013 (2)	0.0004 (19)
C9B	0.037 (3)	0.021 (3)	0.035 (3)	−0.012 (2)	0.026 (2)	−0.012 (2)
C10B	0.018 (2)	0.038 (3)	0.021 (2)	0.010 (2)	0.008 (2)	0.008 (2)
C11B	0.025 (2)	0.016 (2)	0.021 (2)	0.006 (2)	0.012 (2)	0.0010 (18)
C12B	0.027 (2)	0.011 (2)	0.021 (2)	0.002 (2)	0.011 (2)	−0.0016 (18)
C13B	0.021 (2)	0.020 (2)	0.022 (2)	−0.003 (2)	0.009 (2)	−0.003 (2)
O1WB	0.024 (2)	0.031 (2)	0.053 (3)	−0.0026 (17)	0.017 (2)	−0.022 (2)

### Geometric parameters (Å, º)

**Table:**

Cl1A—C11A	1.754 (5)	Cl1B—C11B	1.751 (5)
O1A—C6A	1.228 (6)	O1B—C6B	1.229 (6)
N1A—C3A	1.336 (7)	N1B—C3B	1.335 (6)
N1A—C2A	1.345 (7)	N1B—C2B	1.346 (6)
N2A—C6A	1.351 (6)	N2B—C6B	1.355 (6)
N2A—N3A	1.385 (5)	N2B—N3B	1.384 (5)
N2A—H1NA	1.0006	N2B—H1NB	0.8938
N3A—C7A	1.286 (6)	N3B—C7B	1.286 (6)
C1A—C5A	1.385 (7)	C1B—C2B	1.386 (7)
C1A—C2A	1.396 (7)	C1B—C5B	1.398 (7)
C1A—H1AA	0.9500	C1B—H1BA	0.9500
C2A—H2AA	0.9500	C2B—H2BA	0.9500
C3A—C4A	1.385 (7)	C3B—C4B	1.368 (7)
C3A—H3AA	0.9500	C3B—H3BA	0.9500
C4A—C5A	1.388 (7)	C4B—C5B	1.384 (7)
C4A—H4AA	0.9500	C4B—H4BA	0.9500
C5A—C6A	1.508 (6)	C5B—C6B	1.495 (6)
C7A—C8A	1.460 (6)	C7B—C8B	1.475 (7)
C7A—H7AA	0.9500	C7B—H7BA	0.9500
C8A—C13A	1.408 (7)	C8B—C13B	1.395 (7)
C8A—C9A	1.428 (7)	C8B—C9B	1.405 (7)
C9A—C10A	1.342 (7)	C9B—C10B	1.354 (8)
C9A—H9AA	0.9500	C9B—H9BA	0.9500
C10A—C11A	1.390 (7)	C10B—C11B	1.391 (7)
C10A—H10A	0.9500	C10B—H10B	0.9500
C11A—C12A	1.396 (7)	C11B—C12B	1.383 (7)
C12A—C13A	1.379 (7)	C12B—C13B	1.389 (7)
C12A—H12A	0.9500	C12B—H12B	0.9500
C13A—H13A	0.9500	C13B—H13B	0.9500
O1WA—H1WA	0.89 (8)	O1WB—H1WB	0.81 (9)
O1WA—H2WA	0.86 (9)	O1WB—H2WB	0.73 (7)
			
C3A—N1A—C2A	117.1 (4)	C3B—N1B—C2B	116.9 (4)
C6A—N2A—N3A	118.6 (4)	C6B—N2B—N3B	116.8 (4)
C6A—N2A—H1NA	118.9	C6B—N2B—H1NB	115.7
N3A—N2A—H1NA	121.9	N3B—N2B—H1NB	127.6
C7A—N3A—N2A	114.9 (4)	C7B—N3B—N2B	116.2 (4)
C5A—C1A—C2A	118.4 (5)	C2B—C1B—C5B	119.0 (4)
C5A—C1A—H1AA	120.8	C2B—C1B—H1BA	120.5
C2A—C1A—H1AA	120.8	C5B—C1B—H1BA	120.5
N1A—C2A—C1A	123.1 (5)	N1B—C2B—C1B	122.7 (4)
N1A—C2A—H2AA	118.5	N1B—C2B—H2BA	118.7
C1A—C2A—H2AA	118.5	C1B—C2B—H2BA	118.7
N1A—C3A—C4A	124.0 (5)	N1B—C3B—C4B	124.6 (4)
N1A—C3A—H3AA	118.0	N1B—C3B—H3BA	117.7
C4A—C3A—H3AA	118.0	C4B—C3B—H3BA	117.7
C3A—C4A—C5A	118.2 (5)	C3B—C4B—C5B	118.6 (4)
C3A—C4A—H4AA	120.9	C3B—C4B—H4BA	120.7
C5A—C4A—H4AA	120.9	C5B—C4B—H4BA	120.7
C1A—C5A—C4A	119.1 (4)	C4B—C5B—C1B	118.2 (4)
C1A—C5A—C6A	124.6 (4)	C4B—C5B—C6B	119.1 (4)
C4A—C5A—C6A	116.4 (4)	C1B—C5B—C6B	122.7 (4)
O1A—C6A—N2A	123.9 (4)	O1B—C6B—N2B	123.9 (4)
O1A—C6A—C5A	120.7 (4)	O1B—C6B—C5B	120.0 (4)
N2A—C6A—C5A	115.3 (4)	N2B—C6B—C5B	116.1 (4)
N3A—C7A—C8A	121.0 (4)	N3B—C7B—C8B	119.2 (4)
N3A—C7A—H7AA	119.5	N3B—C7B—H7BA	120.4
C8A—C7A—H7AA	119.5	C8B—C7B—H7BA	120.4
C13A—C8A—C9A	117.1 (4)	C13B—C8B—C9B	118.3 (5)
C13A—C8A—C7A	119.1 (4)	C13B—C8B—C7B	120.5 (4)
C9A—C8A—C7A	123.8 (4)	C9B—C8B—C7B	121.1 (4)
C10A—C9A—C8A	121.8 (4)	C10B—C9B—C8B	121.6 (5)
C10A—C9A—H9AA	119.1	C10B—C9B—H9BA	119.2
C8A—C9A—H9AA	119.1	C8B—C9B—H9BA	119.2
C9A—C10A—C11A	119.4 (5)	C9B—C10B—C11B	119.0 (4)
C9A—C10A—H10A	120.3	C9B—C10B—H10B	120.5
C11A—C10A—H10A	120.3	C11B—C10B—H10B	120.5
C10A—C11A—C12A	121.8 (4)	C12B—C11B—C10B	121.6 (4)
C10A—C11A—Cl1A	119.1 (4)	C12B—C11B—Cl1B	119.4 (4)
C12A—C11A—Cl1A	119.1 (3)	C10B—C11B—Cl1B	119.1 (4)
C13A—C12A—C11A	118.1 (4)	C11B—C12B—C13B	118.7 (4)
C13A—C12A—H12A	120.9	C11B—C12B—H12B	120.7
C11A—C12A—H12A	120.9	C13B—C12B—H12B	120.7
C12A—C13A—C8A	121.7 (4)	C12B—C13B—C8B	120.7 (4)
C12A—C13A—H13A	119.1	C12B—C13B—H13B	119.6
C8A—C13A—H13A	119.1	C8B—C13B—H13B	119.6
H1WA—O1WA—H2WA	114 (8)	H1WB—O1WB—H2WB	111 (8)
			
C6A—N2A—N3A—C7A	−178.2 (4)	C6B—N2B—N3B—C7B	−176.3 (4)
C3A—N1A—C2A—C1A	−2.4 (7)	C3B—N1B—C2B—C1B	−0.6 (7)
C5A—C1A—C2A—N1A	1.4 (7)	C5B—C1B—C2B—N1B	0.4 (7)
C2A—N1A—C3A—C4A	0.6 (7)	C2B—N1B—C3B—C4B	0.2 (7)
N1A—C3A—C4A—C5A	2.1 (7)	N1B—C3B—C4B—C5B	0.3 (8)
C2A—C1A—C5A—C4A	1.3 (7)	C3B—C4B—C5B—C1B	−0.5 (7)
C2A—C1A—C5A—C6A	−179.1 (4)	C3B—C4B—C5B—C6B	−178.7 (4)
C3A—C4A—C5A—C1A	−3.0 (7)	C2B—C1B—C5B—C4B	0.1 (7)
C3A—C4A—C5A—C6A	177.4 (4)	C2B—C1B—C5B—C6B	178.2 (4)
N3A—N2A—C6A—O1A	−0.6 (7)	N3B—N2B—C6B—O1B	0.4 (7)
N3A—N2A—C6A—C5A	177.3 (4)	N3B—N2B—C6B—C5B	179.6 (4)
C1A—C5A—C6A—O1A	−155.3 (4)	C4B—C5B—C6B—O1B	24.6 (7)
C4A—C5A—C6A—O1A	24.3 (6)	C1B—C5B—C6B—O1B	−153.5 (5)
C1A—C5A—C6A—N2A	26.7 (6)	C4B—C5B—C6B—N2B	−154.6 (4)
C4A—C5A—C6A—N2A	−153.8 (4)	C1B—C5B—C6B—N2B	27.3 (6)
N2A—N3A—C7A—C8A	−179.6 (4)	N2B—N3B—C7B—C8B	−178.9 (4)
N3A—C7A—C8A—C13A	176.6 (4)	N3B—C7B—C8B—C13B	169.7 (4)
N3A—C7A—C8A—C9A	−3.9 (7)	N3B—C7B—C8B—C9B	−8.5 (7)
C13A—C8A—C9A—C10A	−0.3 (7)	C13B—C8B—C9B—C10B	−1.5 (7)
C7A—C8A—C9A—C10A	−179.8 (4)	C7B—C8B—C9B—C10B	176.7 (4)
C8A—C9A—C10A—C11A	−0.1 (7)	C8B—C9B—C10B—C11B	0.1 (7)
C9A—C10A—C11A—C12A	0.2 (7)	C9B—C10B—C11B—C12B	1.6 (7)
C9A—C10A—C11A—Cl1A	178.8 (4)	C9B—C10B—C11B—Cl1B	−178.5 (4)
C10A—C11A—C12A—C13A	0.1 (7)	C10B—C11B—C12B—C13B	−1.9 (7)
Cl1A—C11A—C12A—C13A	−178.5 (4)	Cl1B—C11B—C12B—C13B	178.2 (4)
C11A—C12A—C13A—C8A	−0.5 (7)	C11B—C12B—C13B—C8B	0.4 (7)
C9A—C8A—C13A—C12A	0.6 (7)	C9B—C8B—C13B—C12B	1.2 (7)
C7A—C8A—C13A—C12A	−179.9 (5)	C7B—C8B—C13B—C12B	−177.0 (4)

### Hydrogen-bond geometry (Å, º)

**Table:**

*D*—H···*A*	*D*—H	H···*A*	*D*···*A*	*D*—H···*A*
N2*A*—H1*NA*···O1*WA*	1.00	1.88	2.838 (7)	160
N2*B*—H1*NB*···O1*WB*^i^	0.89	1.95	2.810 (7)	161
O1*WA*—H1*WA*···N1*A*^ii^	0.88 (9)	2.14 (8)	2.896 (7)	144 (6)
O1*WA*—H2*WA*···O1*B*^iii^	0.86 (10)	2.05 (9)	2.817 (6)	149 (9)
O1*WA*—H2*WA*···N3*B*^iii^	0.86 (10)	2.59 (10)	3.306 (6)	142 (8)
O1*WB*—H2*WB*···O1*A*^iv^	0.73 (9)	2.19 (8)	2.843 (6)	150 (8)
O1*WB*—H1*WB*···N1*B*^iv^	0.81 (9)	2.00 (9)	2.798 (6)	166 (11)
C1*A*—H1*AA*···O1*WA*	0.95	2.49	3.321 (6)	146
C1*A*—H1*AA*···O1*B*^iii^	0.95	2.54	3.277 (7)	135
C7*A*—H7*AA*···O1*WA*	0.95	2.46	3.247 (7)	141
C1*B*—H1*BA*···O1*WB*^i^	0.95	2.43	3.201 (7)	138
C1*B*—H1*BA*···O1*A*^v^	0.95	2.52	3.230 (8)	131

Symmetry codes: (i) *x*−1, *y*, *z*; (ii) −*x*+1, *y*+1/2, −*z*+3/2; (iii) −*x*+1, −*y*+1, −*z*+2; (iv) −*x*+1, *y*−1/2, −*z*+3/2; (v) −*x*, *y*−1/2, −*z*+3/2.
